# Bone Alterations in Inflammatory Bowel Diseases: Role of Osteoprotegerin

**DOI:** 10.3390/jcm11071840

**Published:** 2022-03-26

**Authors:** Kateryna Priadko, Antimo Moretti, Giovanni Iolascon, Antonietta Gerarda Gravina, Agnese Miranda, Dolores Sgambato, Cristiana De Musis, Marco Romano, Francesca Gimigliano

**Affiliations:** 1 Department of Precision Medicine and Hepato-Gastroenterology Unit, University Hospital and Università degli Studi della Campania “Luigi Vanvitelli”, 80138 Naples, Italy; kateryna.priadko@unicampania.it (K.P.); antoniettagerarda.gravina@unicampania.it (A.G.G.); mirandaagnese@gmail.com (A.M.); dolores.sgambato@gmail.com (D.S.); cristiana.demusis@gmail.com (C.D.M.); marco.romano@unicampania.it (M.R.); 2 Department of Medical and Surgical Specialties and Dentistry, University of Campania “Luigi Vanvitelli”, 80138 Naples, Italy; giovanni.iolascon@gmail.com; 3 Department of Physical and Mental Health, University of Campania “Luigi Vanvitelli”, 80138 Naples, Italy; francesca.gimigliano@unicampania.it

**Keywords:** inflammatory bowel disease, osteoprotegerin, osteoporosis

## Abstract

Metabolic bone disorders are one of the most frequent extra-intestinal manifestations in patients with inflammatory bowel diseases (IBD) that might result in an increase of skeletal fragility and risk of fracture. These disorders are a consequence of bone–gut crosstalk alterations, particularly due to inflammation, which involves the RANK-RANKL-Osteoprotegerin (OPG) pathway. This cross-sectional study investigates the role of serum OPG on bone health in IBD patients. In all patients, we carried out BMD measurements at the lumbar spine and femoral neck by the dual-energy X-ray absorptiometry (DXA), and evaluation of serum OPG, 25(OH)D, and PTH. We also divided all IBD patients into two groups: group 1 consisted of premenopausal women and men younger than 50 years old, while group 2 included postmenopausal women and men aged more than 50 years old. We enrolled 36 UC patients (51%), 34 CD patients (49%), and 70 healthy controls. IBD group mean age was 44 ± 17.3 years old, with a mean disease duration of 6 years. IBD patients had a mean value of OPG of 48.1 ± 26.64 pg/mL, while mean OPG in the control group was 61.35 ± 47.19 pg/mL (*p* < 0.05). In group 1, there was a correlation between BMD Z-scores at the lumbar spine and femoral neck and mean OPG levels in UC subjects (r = 0.47 and r = −0.21, respectively; *p* < 0.05), and only between Z-score at the lumbar spine and OPG level in the CD group (r = 0.83, *p* < 0.05). For the patients of group 2, we report a statistically significant correlation between T-score measured at the lumbar site in both UC and CD patients (r = −0.79 and r = 0.77, respectively; *p* < 0.05). In our study, we demonstrated serum OPG levels to be significantly decreased in IBD subjects compared to healthy age-matched individuals. However, according to our data, it seems that the measurement of serum OPG levels is not useful to better define metabolic bone disorders in IBD patients.

## 1. Introduction

Inflammatory bowel disease (IBD) is a group of chronic intestinal disorders that are typically categorized as one of two subtypes: Crohn’s disease and ulcerative colitis. Ulcerative colitis is limited to the colon, with superficial mucosal inflammation that extends continuously in the proximal direction and can be complicated by ulcers, severe bleeding, toxic megacolon, and fulminant colitis. Crohn’s disease, on the contrary, can affect any part of the digestive tube, often can be described by skip lesions, and is characterized by transmural inflammation, which can cause complications such as fibrotic strictures, fistulas, and abscesses [1,2,3]. Of note, up to 50% of patients with IBD experience at least one extra-intestinal manifestation (EIM), which can present before IBD is diagnosed and can possibly involve any organ system [4,5,6,7]. Metabolic bone disorders (i.e., osteopenia and osteoporosis) are one of the most frequent EIMs in IBD patients [1,8]. These conditions may heavily influence disability, quality of life, and costs of management of IBD patients. IBD-related metabolic bone disorders are typically characterized by an increase in osteoclast activity and bone resorption, but also a reduced bone formation can occur. Both mechanisms cause a reduction of quantity and alterations of quality of bone tissue, that might result in an increase of skeletal fragility and risk of fracture. These disorders are a consequence of bone–gut crosstalk alterations, since the gastrointestinal (GI) tract may communicate with bone through blood, nerves, and immune cells, defining a characteristic gut-to-bone signaling axis that also involves incretins, serotonin, and GI microbiota [1,9,10].

Among regulating factors of bone metabolism, the vitamin D system, a key modulator of calcium-phosphate homeostasis and bone turnover, might be adversely affected in IBD patients by multiple factors [11]. Indeed, vitamin D deficiency in this population [12] might occur because of reduced dietary intake, malabsorption related to the small intestine disease, short bowel syndrome secondary to small intestine resection, or development of entero-enteric fistulae. Gastrointestinal loss of other nutrients may also contribute to IBD-related bone loss as a consequence of malabsorption and diarrhea. Moreover, inflammation may cause mucosal ulceration and chronic blood loss with protein loss within the intestinal lumen [13] and often leads to the need of recurrent corticosteroid (CS) treatment that could affect bone health [14]. Without any doubt, the main contributor to the alteration of bone metabolism is inflammation, which causes an overexpression of molecules modulating osteoclasts’ activity [15]. Among these, the RANK-RANKL-Osteoprotegerin (OPG) pathway has been described as a key factor in the regulation of bone turnover. RANKL is a member of the TNF ligand family secreted by osteoblasts, which binds the activating NfKB receptor called RANK, expressed on osteoclast lineage. The RANKL–RANK interaction allows osteoclast differentiation and osteoclastogenesis. Osteoprotegerin is produced by osteoblasts and competes with the RANKL for binding to the RANK receptor, thus blocking osteoclastogenesis. The RANKL:OPG ratio is a cornerstone of bone remodeling. Many factors, such as parathyroid hormone (PTH), prostaglandin E2 (PGE2), and 25(OH)D, can increase the expression of RANKL and can inhibit the OPG production. In the IBD population, it has been observed that the alteration of the RANKL:OPG ratio in favor of RANKL could play a role in bone loss [13]. This study was aimed at investigating the role of serum OPG levels on bone health in IBD patients.

## 2. Materials and Methods

### 2.1. Study Design and Participants

We conducted a case-control study between January 2017 and June 2019. Inclusion criteria for the study group were a well-established diagnosis of IBD and age ≥ 18 and ≤65 years. Exclusion criteria were age <18 or >65, pregnancy, and diagnosis of acquired or congenital immunodeficiencies. Inclusion criteria for the control group were healthy subjects, without GI, inflammatory, or rheumatic diseases, and aged ≥18 or ≤65 years. All data were collected using a card for the recording of personal data and for the collection of anamnestic and pharmacological data, as well as those relating to laboratory and instrumental examinations. When calculating the number of steroid cycles, one steroid cycle was defined as the exposure to conventional oral glucocorticoid therapy at a starting dose of 0.75–1 mg/kg oral prednisone-equivalent and succeeding tapering for 4–6 weeks, with daily dosage lowered by 5–10 mg every week, while prolonged CS use was considered as that of total duration exceeding 3 months [16,17]. The measurement of serum OPG was not performed while on steroid therapy and at least 10 days after the last steroid therapy dose received. In all IBD patients, regional BMD at the lumbar spine (LS; L1–L4 tract) and femoral neck (FN) were measured by the dual-energy X-ray absorptiometry (DXA) for the diagnosis of low bone mass or osteoporosis, according to international guidelines [18,19]. Osteoporotic fractures were considered as those that resulted from “low-energy” trauma which ordinarily would not cause a fracture [18]. At the next step, all IBD patients underwent blood sampling for the evaluation of serum levels of OPG, 25(OH)D, and PTH, while the control group was subjected to the measurement of serum OPG levels only. We also divided all IBD patients into two groups according to the guidelines for the definition of osteoporosis: group 1 consisted of premenopausal women and men younger than 50 years old, while group 2 included postmenopausal women and men aged more than 50 years old [18,19]. This study was approved by the ethical committee of the department of Precision Medicine of the University of Campania “Luigi Vanvitelli” (5 January 2017, DPM7/2017). All subjects gave their consent to participate in this study. In our informed consent for patients undergoing a clinical study, it is clearly stated: “I agree to the publication of data regarding this study. I am fully aware of the implications of publication and accept any associated risk”.

### 2.2. Bone Mineral Density Measurement: Dual X-ray Absorptiometry

We tested the bone loss by measuring BMD (g/cm^2^) using DXA. BMD is determined by a planar measurement of X-ray extinction [20]. The operational definition of osteoporosis for postmenopausal women and men >50 years old is at the T-score ≤ −2.5 standard deviations (SD). Severe osteoporosis was established by a BMD T-score ≤ −2.5 SD if associated with a fragility fracture. T-score values between −1 and −2.5 defined low bone mass or osteopenia. T-score is expressed as the number of SD that differentiate the subject examination from the mean BMD value observed in a reference population composed of young adults of the same sex. In premenopausal women and men younger than 50 years old, the parameter to consider is the Z-score, which is the number of SD that differentiate the subject examination from a reference population of the same gender, age, and ethnicity. In this group, if the Z-score was ≤−2.0 SD, the term “low bone mass” or “low bone density” was used [18,21].

### 2.3. Laboratory Methods

#### 2.3.1. OPG Serum Level: ELISA Assay

OPG serum level was detected using a commercial Human Osteoprotegerin enzyme-linked immunosorbent assay (ELISA kit—Abcam, Cambridge, UK). Each whole-blood sample was centrifuged. Plasma samples were loaded into microplates pre-coated with specific anti-OPG monoclonal antibodies and both standards and samples were bound by immobilized antibodies. Subsequently, two washings were performed, and an OPG-specific polyclonal antibody was added to each well. The sensitivity of the kit is 1 pg/mL, range 1.23–900 pg/mL. The intra-assay precision of the kit, measured as coefficients of variation (CV%), was reported as <10%, while inter-assay was <12%. We report the levels of OPG in the form of mean ± SD in pg/mL.

#### 2.3.2. Serum 25-Hydroxyvitamin D 

The measurement of serum 25(OH)D was performed in the morning after 8–12 h of fasting via solid-phase enzyme-linked immunoassay (ELISA) based on the principal of competitive binding. Blood samples were collected by venipuncture, centrifuged, and the plasma was stored at −20 °C until the number needed to perform the test was reached. For the assay, the commercial 25(OH)D Vitamin D ELISA kit (Abcam, Cambridge, UK) was used. Dissociation buffer was added to wells coated with donkey anti-sheep IgG antibody. Standards and samples were then added to these wells. Then, the solution of alkaline phosphatase conjugated 25(OH)D and sheep monoclonal antibody were added to 25(OH)D. During incubation, the antibody was captured by the anti-sheep IgG antibody. A pNpp substrate solution was added that generated a yellow color in the solution. Stop solution was added to stop the substrate reaction and the resulting yellow color was read at 405 nm. The amount of signal was inversely proportional to the level of 25(OH)D in the sample. The results appear in ng/mL. The biological sensitivity of the assay was 1.98 ng/mL. The sensitivity was determined by interpolation at 2 SD below the mean signal at a concentration of 0 ng/mL analyte (*n* = 20) using data from 25 standard curves. Intra-assay precision varied from 1.6% to 3.4%, while inter-assay precision for 2 sample groups was estimated to vary from 11.5% to 15.8%, according to the 25(OH)D ELISA Kit (ab213966) protocol booklet. The optimal 25(OH)D level is controversial: AACE and the Endocrine Society recommend serum 25(OH)D ≥30 ng/mL to define vitamin D sufficiency, and our study protocol was guided by this recommendation [22].

#### 2.3.3. Serum Parathyroid Hormone (PTH)

Blood samples were collected by venipuncture, centrifuged, and the plasma was stored at −20 °C until the assay was performed. The recombinant anti-parathyroid antibody kit for sandwich ELISA (Abcam, Cambridge, UK) was used. The results appear in pg/mL. Physiological range: 15–65 pg/mL.

### 2.4. Statistical Analysis 

Statistical analysis was performed using the GraphPad Prism 8 Software package. We report continuous data as mean values ± SD. Categorical variables are presented as frequency counts with percentages. For the analysis of all of our data with normal distribution, we used parametric tests (Student’s *t*-test or Pearson’s correlation test). Statistical significance was defined by a *p*-value < 0.05.

## 3. Results

### 3.1. Subjects’ Characteristics

In this study, we enrolled 36 UC patients (51%), 34 CD patients (49%), and 70 healthy controls. Study group patients and the control group were comparable in age and sex distribution. IBD group average age was 44 ± 17.3 years old, while that of the control group was 46 ± 20.1 years old. Male/female (M/F) ratios were 1.25:1 and 1.12:1, correspondingly. There was a notable mean age difference between males and females in the UC group (50 vs. 39 years old). Average disease duration for patients with IBD was 6 years. Average steroid cycles were 3.7 (Table 1). According to the division of IBD patients into two groups for the evaluation of BMD and the bone metabolism, group 1 included 40 subjects and group 2 consisted of 30 (Table 2). 

### 3.2. Serum Osteoprotegerin Level

We report a mean value of OPG in the IBD group of 48.1 ± 26.64 pg/mL, while mean OPG in the control group was 61.35 ± 47.19 pg/mL (*p* < 0.05). Mean OPG had a tendency (not statistically significant) to be lower in men in both IBD subgroups compared to that of women: 49.9 vs. 51.4 pg/mL for UC (*p* > 0.05) and 41.7 vs. 53.7 pg/mL for CD (*p* > 0.05) (Table 3 and Table 4). Moreover, we found a statistically significant weak correlation between age and OPG level (r = 0.33, *p* < 0.05) in IBD patients. Interestingly, in group 1, there was a correlation between BMD Z-scores at the lumbar spine and femoral neck and mean OPG levels in UC subjects (r = 0.47 and r = −0.21, respectively; *p* < 0.05), and only between Z-score at the lumbar spine and OPG level in the CD group (r = 0.83, *p* < 0.05) (Table 4). For the patients of group 2, we report a statistically significant correlation between T-score measured at the lumbar site in both UC and CD patients (r = −0.79 and r = 0.77, respectively; *p* < 0.05) (Table 4).

### 3.3. Bone Mineral Density in IBD Patients

BMD loss (Z-score ≤ 2.0 SD) was reported in 16/40 (40%) patients from group 1, and none of them had fragility fractures. In this group, there were 19/40 (47.5%) premenopausal women and 21/40 (52.5%) men under the age of 50 (Table 5). Among the patients of group 2, BMD alterations were noted in 21/30 (70%) subjects. Out of the 30 patients, 13 (43%) had osteopenia and 8 (27%) had osteoporosis, and in 3 of those with BMD alterations, there was a history of multiple fractures (Table 6). Sex distribution in the second group was as follows: 12 (40%) women in menopause and 18 (60%) males over 50 years. Lumbar spine and FN Z-scores in patients from group 1 were comparable for both subgroups of UC and CD: −0.94 and −0.97 (*p* > 0.05) at LS and −0.86 and −0.88 at FN, respectively (*p* > 0.05) (Table 4). We found that patients of group 2 with UC had significantly higher mean T-scores at LS compared to CD patients, while such comparison at FN was not found to be significant: −0.44 vs. −1.35 (*p* < 0.05) and −1.38 vs. −1.92 (*p* > 0.05) at LS and FN, respectively (Table 4).

### 3.4. Serum Vitamin D

All enrolled IBD patients as well as the control group carried out the serum 25(OH)D dosage. We report the mean values of 24.16 (±8.9) ng/mL for the IBD group and 35.6 (±11.4) ng/mL for the control group (physiological range 30–100 ng/mL). In the IBD group, 32 out of 70 patients (45.7%) had normal serum 25(OH)D; among patients with normal serum 25(OH)D, there were 20 patients from group 1 and 12 from group 2 (Table 7 and Table 8). Only in the second group was the prevalence of low bone mass noticeable in patients with low serum 25(OH)D compared to those with normal serum 25(OH)D: 10/40 (25%) vs. 6/40 (15%) (*p* > 0.05) patients in the first group and 16/30 (53%) vs. 8/30 (27%) (*p* < 0.05) in the second group, respectively (Table 7 and Table 8). Moreover, a statistically significant correlation was found between mean BMD T-score and serum 25(OH)D levels (r = 0.42, *p* < 0.05) in patients from the second group. We also report that all three patients with fragility fracture history had vitamin D deficiency, although the correlation between serum 25(OH)D and BMD resulted not statistically significant (*p* = 0.3).

### 3.5. Serum Parathyroid Hormone

A serum PTH value was provided by all patients, of which 10/70 (15%) had a lower value than the physiological range, and 2/70 (4%) had PTH hypersecretion.

We would like to mention that among the two patients with PTH hypersecretion, both of them had hypocalciuria (<100 mg/day), they were both male, one was a patient with UC, while another one was affected with CD. Notably, both patients had low 25(OH) vitamin D levels (serum level < 20 ng/mL).

## 4. Discussion

In our study, we demonstrated serum OPG levels to be significantly decreased in IBD subjects compared with healthy age-matched individuals. However, the results of previous studies aiming at measuring OPG levels in IBD patients to define its role in bone metabolism are conflicting. Moschen et al. showed serum OPG to be generally increased in IBD patients [13], while a Polish study reported the highest serum OPG levels in the CD group [15], whereas Bernstein et al. reported OPG to be significantly higher only in CD-affected females [23]. A study group from Czech Republic also confirmed higher levels of OPG in the IBD population, but lower serum OPG in those receiving anti-TNF-α treatment [24]. On the other hand, another study aiming to investigate OPG gene polymorphism in IBD patients reported that mean serum OPG in CD patients did not significantly differ from controls, whereas in UC patients, OPG levels were significantly lower [25]. 

A hypothesis for such confounding results is that OPG expression is the final step of different biological pathways regulating osteoblasts’ activity, including Wnt/β-catenin. Additionally, in osteoclasts, the OPG:RANKL expression ratio is modulated by other signaling, such as Jagged1/Notch1, regulating osteoclastogenesis [26,27]. Moreover, OPG is likely to exert its pro-inflammatory effects through RANK activation and may contribute to IBD pathogenesis [28]. At the same time, it was reported that anti-TNF treatment exerts potential anti-inflammatory effects by dramatically lowering serum OPG, increasing osteocalcin (marker of bone formation) and reducing bone resorption markers [29]. Miheller et al. described that anti-TNF agents (Infliximab) significantly decreased the OPG concentration in CD patients, thus suggesting that elevated OPG in CD could be a counter-regulatory response to inflammatory cytokines or may indicate T-cell activation [29]. On the contrary, Aschroft et al. showed the opposite in bone alterations in a mouse model of colitis (IL-2-deficient mice) after an exogenous administration of OPG, therefore suggesting that OPG is protective against osteoporosis and its possible role as a therapeutic agent [30]. 

Age seems to be a contributing factor to serum OPG increase. In our study, we found a statistically significant weak positive correlation between age and OPG levels, as previously reported by both an Italian study demonstrating OPG to be significantly increased in postmenopausal women compared to fertile age women [31] and by an Icelandic study that demonstrated a significant association between age and OPG in a random sample of community-dwelling adults [32]. 

Typically, bone damage in different populations is investigated by BMD measurements. We found BMD alterations in 57.1% of all the IBD subjects with a lower BMD T-score in CD compared to UC patients at LS (*p* < 0.05). Numerous studies also highlighted that patients with CD vs. those with UC have both higher prevalence of osteoporosis as well as lower mean BMD values, probably because of the higher frequency of small-bowel disease or resection, smoking, and CS treatment in CD patients [14,33]. Interestingly, in patients aged over 50, we found a strong negative correlation between serum OPG and T-score at LS in subjects affected with UC, while this correlation was equally strong, but positive, in CD patients (r = −0.79, *p* < 0.05 and r = 0.77, *p* < 0.05, correspondingly). Such discrepancy may be partially explained by comparatively higher BMD T-values in patients with UC as well as different treatment approaches. In younger patients (group 1), the correlation between bone mass and serum OPG was confirmed in UC (r = −0.5, *p* < 0.05), whereas it resulted not significant in CD patients (r = 0.65, *p* = 0.3). However, most studies investigating the association between OPG and BMD failed to prove a significant association between these parameters [32]. 

We would like to disclose some limitations of our study. First of all, we did not systematically evaluate the CS use as well as disease duration by IBD patients, which did not allow us to analyze its relationship with other important parameters, i.e., BMD and OPG. Similarly, the number of patients taking oral vitamin D supplements was not recorded, which could have been analyzed in the relevance to BMD and OPG levels. Secondly, the treatment regimens (including current anti-TNF therapy) were not consistently recorded, which limited the evaluation of serum OPG fluctuation depending on anti-TNF treatment. We would like to mention that the Abcam kit used in our laboratory for 25(OH)D measurement is used for research purposes only, although its sensitivity is comparable to tests approved for clinical use, such as: Liaison 25(OH)D Total test (DiaSorin Liaison XL) (DiaSorin, Saluggia, Italy), Elecsys Vitamin D Total II test (Roche Elecsys) (Roche Diagnostics Gmbh, Mannheim, Germany), and Lumipulse G 25OH Vitamin D test (Fujirebio Lumipulse G1200) (Fujirebio inc, Tokyo, Japan). The coefficients of variation for these tests were 5.1–6.99%, 6.35–11.41%, and 4.32–4.45%, respectively [34]. Finally, we did not measure serum RANKL, thus limiting the understanding of OPG changes in our population. These parameters need to be evaluated in further prospective studies.

## 5. Conclusions

IBD patients are at increased risk of developing osteoporotic changes due to systemic inflammation, CS use, and malabsorption of vitamin D from intestinal lumen due to local inflammation. Such patients need to be thoroughly evaluated for the presence of metabolic bone disorders. According to our data, it seems that the routine measurement of serum OPG levels is not useful to better define metabolic bone disorders in IBD patients. However, OPG might be a key factor in the pathogenesis of bone loss in IBD patients considering its involvement during the activation of systemic inflammation. The role of anti-TNF treatment on serum OPG changes might partially justify their efficacy in the management of bone damage in this population, but this hypothesis requires corroboration by further studies.

## Figures and Tables

**Table 1:** Baseline characteristics of IBD patients and controls.

All IBD Patients	Subjects Enrolled, N (% Total)	Mean Age (Years ± SD)	Steroid Use (N of Cycles/Subject)
	70 (100%)	44 (±17.3)	3.7
CD	34 (49%)	43 (±16.4)	4
-male	18	44 (±16.8)	4.1
-female	16	41 (±16.3)	3.9
UC	36 (51%)	45 (±18.4)	3.4
-male	21	50 (±19.7)	3.4
-female	15	39 (±14.8)	3.3
Control group	70 (100%)	46 (±22.1)	0
-male	37	49 (±20)	0
-female	33	43 (±24.8)	0

Abbreviations: IBD: inflammatory bowel diseases; CD: Crohn’s disease; UC: ulcerative colitis.

**Table 2:** Patients’ distribution among group 1 and 2 according to the osteoporosis definition by age and menopausal state.

	Group 1 (n = 40)	Group 2 (n = 30)
CD	20	14
UC	20	16

Abbreviations: CD: Crohn’s disease; UC: ulcerative colitis.

**Table 3:** Serum OPG levels across each group.

	OPG Mean Level (pg/mL) ± SD	*p*-Value		
All IBD patients	48.1 (±26.64)			*p* < 0.05
CD	47.39 (±24.39)		*p* = 0.8266	
-males	41.7 (±12.77)	*p* = 0.155		
-females	53.7 (±32.29)			
UC	48.8 (±28.9)			
-males	49.9 (±27.67)	*p* = 0.88		
-females	51.4 (±31.43)			
Control group	61.3 (±47.19)			

Abbreviations: IBD: inflammatory bowel diseases; CD: Crohn’s disease; UC: ulcerative colitis. The variables were normally distributed. For the statistical analysis, the Student’s *t*-test was used.

**Table 4:** Correlation between mean BMD T- and Z-scores (±SD) and mean OPG levels for UC and CD in both IBD groups.

UC				CD			
Group 1		Group 2		Group 1		Group 2	
Z-Score Lumbar Spine	Z-Score Femoral Neck	T-Score Lumbar Spine	T-Score Femoral Neck	Z-Score Lumbar Spine	Z-Score Femoral Neck	T-Score Lumbar Spine	T-Score Femoral Neck
−0.94 (±0.98)	−0.86 (±0.91)	−0.44 (±0.82)	−1.38 (±0.89)	−0.97 (±1.05)	−0.88 (±0.96)	−1.35 (±1.54)	−1.92 (±0.94)
Mean OPG ± SD							
59 ± 29.5		43 ± 27.6		54.9 ± 20.3		44.2±25.6	
r value, *p*-value							
r = 0.47, *p* < 0.05	r = −0.5, *p* < 0.05	r = −0.79, *p* < 0.05	r = 0.13, *p* = 0.6	r = 0.83, *p* < 0.05	r = 0.65 *p* = 0.3	r = 0.77, *p* < 0.05	r = −0.48 *p* = 0.08

Abbreviations: CD: Crohn’s disease; UC: ulcerative colitis; Osteoprotegerin (OPG); SD: standard deviations. The variables were normally distributed. For the statistical analysis, Pearson’s correlation test was used.

**Table 5:** Distribution of BMD Z-value alterations among patients with UC and CD of group 1.

	UC (n 20)	CD (n 20)	*p*-Value
Lumbar spine Z-score ≤ −2 SD	5/20 (25%)	7/20 (35%)	*p* = 0.7
Femoral neck Z-score ≤ −2 SD	2/20 (10%)	4/20 (20%)	*p* = 0.8
Lumbar spine Z-score > −2 SD	15/20 (75%)	13/20 (65%)	*p* = 0.5
Femoral neck Z-score > −2 SD	18/20 (90%)	16/20 (80%)	*p* = 0.16
Fragility fractures	0 (0%)	0 (0%)	-

Abbreviations: CD: Crohn’s disease; UC: ulcerative colitis; SD: standard deviations. The variables were normally distributed. For the statistical analysis, the Student’s *t*-test was used.

**Table 6:** Distribution of BMD T-score alterations among patients with UC and CD of group 2.

	UC (n 16)	CD (n 14)	*p*-Value
T-score lumbar spine >−1	5/16 (31%)	4/14 (28.5%)	*p* = 0.8
T-score femoral neck >−1	3/16 (19%)	2/14 (14%)	*p* = 0.6
T-score lumbar spine −1–−2.5 SD	8/16 (50%)	5/14 (36%)	*p* = 0.2
T-score femoral neck −1–−2.5 SD	4/16 (25%)	4/14 (28.5%)	*p* = 0.4
T-score lumbar spine ≤2.5 SD	2/16 (12.5%)	3/14 (21%)	*p* = 0.8
T-score femoral neck ≤2.5 SD	3/16 (19%)	5/14 (36%)	*p* = 0.5
Fragility fractures	2/16 (12.5%)	1/14 (7%)	NS

Abbreviations: CD: Crohn’s disease; UC: ulcerative colitis; SD: standard deviations; NS: not significant. The variables were normally distributed. For the statistical analysis, the Student’s *t*-test was used.

**Table 7:** The prevalence of BMD Z-score value alterations in patients with normal and decreased levels of serum 25(OH)D (group 1).

Serum 25(OH)D—Group 1			
>30 ng/mL		<30 ng/mL	
20 (50%)		20 (50%)	
Z > −2.0	Z ≤ −2.0	Z > −2.0	Z ≤ −2.0
14 (70%)	6 (30%)	10 (50%)	10 (50%)
r = 0.41, *p* = 0.07			

Abbreviation: 25(OH)D: 25-hydroxyvitamin D. The variables were normally distributed. For the statistical analysis, Pearson’s correlation test was used.

**Table 8:** The prevalence of BMD T-score alterations in patients with normal and decreased levels of serum 25(OH)D (group 2).

Serum 25(OH)D—Group 2			
>30 ng/mL		<30 ng/mL	
12 (40%)		18 (60%)	
T > −1	T < −1	T > −1	T < −1
4 (33.3%)	8 (66.6%)	2 (11%)	16 (89%)
r = 0.42, *p* < 0.05			

Abbreviation: 25(OH)D: 25-hydroxyvitamin D. The variables were normally distributed. For the statistical analysis, Pearson’s correlation test was used.

## Data Availability

The data presented in this study are available upon reasonable request from the first author.
